# The C-terminal Six Amino Acids of the FNT Channel FocA Are Required for Formate Translocation But Not Homopentamer Integrity

**DOI:** 10.3389/fmicb.2017.01616

**Published:** 2017-08-22

**Authors:** Doreen Hunger, Marie Röcker, Dörte Falke, Hauke Lilie, R. Gary Sawers

**Affiliations:** ^1^Institute of Microbiology, Martin-Luther University Halle-Wittenberg Halle, Germany; ^2^Institute of Biochemistry and Biotechnology, Martin-Luther University Halle-Wittenberg Halle, Germany

**Keywords:** membrane channel, FNT superfamily, formate, formate translocation, C-terminal peptide, homopentamer

## Abstract

FocA is the archetype of the pentameric formate-nitrite transporter (FNT) superfamily of channels, members of which translocate small organic and inorganic anions across the cytoplasmic membrane of microorganisms. The N- and C-termini of each protomer are cytoplasmically oriented. A Y-L-R motif is found immediately after transmembrane helix 6 at the C-terminus of FNT proteins related to FocA, or those with a role in formate translocation. Previous *in vivo* studies had revealed that formate translocation through FocA was controlled by interaction with the formate-producing glycyl-radical enzyme pyruvate formate-lyase (PflB) or its structural and functional homolog, TdcE. In this study we analyzed the effect on *in vivo* formate export and import, as well as on the stability of the homopentamer in the membrane, of successively removing amino acid residues from the C-terminus of FocA. Removal of up to five amino acids was without consequence for either formate translocation or oligomer stability. Removal of a sixth residue (R280) prevented formate uptake by FocA in a strain lacking PflB and significantly reduced, but did not prevent, formate export. Sensitivity to the toxic formate analog hypophosphite, which is also transported into the cell by FocA, was also relieved. Circular dichroism spectroscopy and blue-native PAGE analysis revealed, however, that this variant had near identical secondary and quaternary structural properties to those of native FocA. Interaction with the glycyl radical enzyme, TdcE, was also unaffected by removal of the C-terminal 6 amino acid residues, indicating that impaired interaction with TdcE was not the reason for impaired formate translocation. Removal of a further residue (L279) severely restricted formate export, the stability of the protein and its ability to form homopentamers. Together, these studies revealed that the Y_278_-L_279_-R_280_ motif at the C-terminus is essential for bidirectional formate translocation by FocA, but that L279 is both necessary and sufficient for homopentamer integrity.

## Introduction

The first member of the evolutionarily ancient homopentameric formate-nitrite transporter (FNT) superfamily of channel proteins to be identified was the formate transporter FocA of *Escherichia coli* (Suppmann and Sawers, 1994; Saier et al., 1999). At that time, only two other related membrane proteins, NirC, also from *E. coli* (Peakman et al., 1990) and FdhC from the formate-utilizing methanogenic archaeon *Methanobacterium formicicum* (White and Ferry, 1992), were found in the sequence database. Meanwhile, genome analyses have revealed that the FNT superfamily of anion channels is widespread in bacteria and archaea, with thousands of examples (Waight et al., 2013). FNT proteins are also found in certain single-celled eukarya (Unkles et al., 2011; Marchetti et al., 2015; Wu et al., 2015; Yamano et al., 2015). Many of the microorganisms in which these FNT proteins are found are pathogens (Waight et al., 2010; Czyzewski and Wang, 2012; Lü et al., 2012b; Marchetti et al., 2015; Wu et al., 2015). Moreover, because FNT channels are not found in higher eukarya, this makes them suitable drug targets and recent studies on the PfFNT lactate channel in the malaria parasite *Plasmodium falciparum* has validated this prospect (Golldack et al., 2017; Hapuarachchi et al., 2017). Notwithstanding their future potential importance in helping combat some infectious diseases, the study of these membrane proteins is of broad general interest for our understanding of the biophysical properties of membrane protein folding, the bioenergetics of substrate translocation through these channels and their role in anaerobic metabolism

**FIGURE 1:**
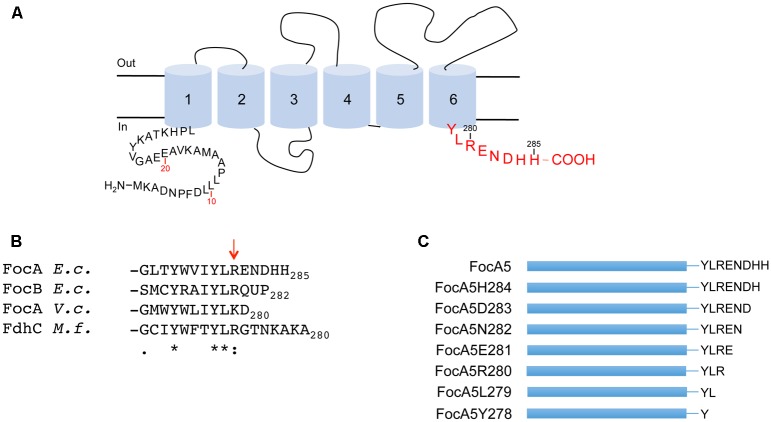
Amino acid sequence and localisation of FocA in the cytoplasmic membrane. **(A)** The schematic organization of a FocA protomer in the membrane is shown. The cytoplasmic N- and C-terminal amino acid residues are shown, along with the transmembrane helices, labeled 1 through 6, and which are represented as blue cylinders. The C-terminal amino acids relevant to this study are shown in red. **(B)** Shows an alignment of the C-terminal amino acids after transmembrane helix 6 of FocA-like proteins belonging to the FNT superfamily. The amino acid sequences include FocA from *Escherichia coli*, FocB from *E. coli* (Andrews et al., 1997), FocA from *Vibrio cholerae* (Waight et al., 2010), and FdhC from *Methanobacterium formicicum* (White and Ferry, 1992). The arrow denotes the location of arginine 280, based on numbering of FocA from *E. coli*. Asterisks highlight invariant amino acids, colons highly conserved and stops conservative substitutions. **(C)** Shows a schematic representation of the FocA5 truncation variants. The blue bar represents the FocA polypeptide and the letters on the right indicate the C-terminal amino acid sequence of the respective variant.

Structural studies have revealed that all FNT channels have a common fold related to that found in the aquaporins (Wang et al., 2009; Waight et al., 2010; Lü et al., 2011; Czyzewski and Wang, 2012; Lü et al., 2012b). Each protomer of the homopentamer is characterized by having six transmembrane helices (**Figure 1A**), which are organized to form an ‘hour-glass’-shaped substrate channel that restricts passage of the substrate across the cytoplasmic membrane and presumably ensures maintenance of a tight seal, preventing dissipation of the proton gradient (Lü et al., 2013; Waight et al., 2013). Both N- and C-termini of each protomer are located in the cytoplasm and are variable both in length and with regard to their amino acid sequences between different FNT members. One short amino acid sequence of Y-L-R/K appears to be conserved in the C-terminus of FocA-related proteins (**Figure 1B**). This sequence is located immediately after the sixth transmembrane helix as it emerges into the cytoplasm (Wang et al., 2009; see **Figure 1A**). The role of the C-terminus in regulating nitrite transport through the NitM FNT protein, which is found in some marine cyanobacteria, has recently been demonstrated (Maeda et al., 2015) and although the amino acid sequence is not conserved at the C-terminus of FocA proteins, charged amino acids, particularly arginines, might influence formate translocation by FocA. Therefore, a major aim of the current study was to determine the importance of the cytoplasmic C-terminus in FocA function.

The substrates translocated by FNT channels currently include only the small anions formate, bicarbonate, nitrite, hydrosulfide and lactate (Czyzewski and Wang, 2012; Lü et al., 2012b; Beyer et al., 2013; Yamano et al., 2015; Wiechert and Beitz, 2017), although *E. coli* FocA can also translocate the formate analog hypophosphite *in vivo*, which restricts fermentative growth of the bacterium by irreversibly inhibiting the glycyl radical enzyme pyruvate formate-lyase (PflB) (Suppmann and Sawers, 1994). There is considerable debate, however, regarding the mechanism of substrate translocation through these channels (Lü et al., 2013; Waight et al., 2013; Wiechert and Beitz, 2017) and although isolated channels translocate a number of anions *in vitro* (Lü et al., 2012a,b), current evidence suggests that these channels are highly substrate-specific *in vivo* (Beyer et al., 2013). Moreover, the direction of substrate translocation in cells is variable and this depends on the metabolism of the host. Thus, while FocA and NirC translocate their respective substrate bi-directionally (Suppmann and Sawers, 1994; Jia et al., 2009; Beyer et al., 2013), others probably function mainly uni-directionally under physiological conditions. For example, based on the metabolic demands of their respective host the HSC hydrosulfide channel in *Clostridium difficile* (Czyzewski and Wang, 2012) and the PfFNT lactate channel in *P. falciparum* probably function primarily in the export direction (Marchetti et al., 2015; Wu et al., 2015), whereas FdhC from *M. formicicum* functions to import the substrate formate for methanogenesis (White and Ferry, 1992). Recent studies have provided evidence for a proton symport-based mechanism for the uptake of lactate by the PfFNT channel from *P. falciparum* when the channel is heterologously synthesized in *Saccharomyces cerevisiae* (Wiechert and Beitz, 2017); however, unimpeded import of substrate would lead to rapid acidification of the cytoplasm, uncoupling of the membrane potential, and ultimately causing cell death. Therefore, both import and export of substrate probably are controlled processes (Lü et al., 2013; Waight et al., 2013).

*In vivo* studies carried out with *E. coli* have established that import of formate by FocA no longer occurs if formate cannot be metabolized (Beyer et al., 2013). This observation suggests a ‘gating’ mechanism to prevent unimpeded substrate entry into cells. Indeed, a subsequent study identified a specific interaction between PflB, the enzyme responsible for generating formate, and the N-terminal helix of FocA (Doberenz et al., 2014). PflB exists in both active and inactive forms (Wagner et al., 1992) and results indicate that in its inactive form PflB impedes formate import (Doberenz et al., 2014). A similar result was observed in mutants lacking PflB (Doberenz et al., 2014; Hunger et al., 2014), suggesting that the active PflB enzyme is necessary to promote formate translocation *in vivo*.

Development of a reporter system based on a formate-responsive promoter (Falke et al., 2010) has greatly facilitated the *in vivo* assessment of both import and export of the anion through FocA (Hunger et al., 2014). This system has also helped to identify amino acids in the central substrate pore of the FocA protomer that are important for channel activity (Hunger et al., 2014). However, no study has yet been carried out to examine the roles of the cytoplasmic N- or C-termini of these proteins and how they impact substrate transport and homopentamer formation. Based on its structure, FocA from *E. coli* has a short, seven amino-acid long C-terminal helix and it is unclear what role this has on channel function. Therefore, in this study we have conducted a detailed analysis of the effect of successive single amino acid truncations at the C-terminus and identify the minimum requirements for formate export and homopentamer stability.

## Materials and Methods

### Strains and Growth Conditions

All bacterial strains and plasmids used in this study are listed in **Table 1**. Strains DH201 and DH701, originally described in (Hunger et al., 2014) are derivatives of RM201 (Δ*focA*Δ*pflB*; Sawers and Böck, 1988) and REK701 (*focA*; Suppmann and Sawers, 1994), respectively, carrying the single-copy λ *fdhF*::*lacZ* fusion. Preparation of aerobic cultures of *E. coli* for molecular biology experiments was performed in Erlenmeyer flasks filled to a maximum of 10% of their volume with Luria–Bertani (LB) medium (Sambrook et al., 1989). Aerobically grown cultures were incubated on a rotary shaker (250 rpm) at 37°C.

**Table 1:** Strains, and plasmids used in this study.

Strain or plasmid	Relevant genotype or characteristic(s)	Reference
Strain		
BL21(DE3)	F^-^ *ompT hsdS*(rB^-^ mB^-^) *gal dcm lacY1* (DE3)	Invitrogen, Carlsbad, CA, United States
MC4100	F^-^ *araD139* (*argF-lac*)*U169 ptsF25 deoC1 relA1 flbB5301 rspL150*	Casadaban, 1976
DH4100	MC4100 λ *fdhF*::*lacZ* Kan^R^	Hunger et al., 2014
DH201	Like RM201 but λ *fdhF*::*lacZ* Kan^R^ Cm^R^	Hunger et al., 2014
DH701	Like REK701 but λ *fdhF*::*lacZ* Kan^R^	Hunger et al., 2014
Plasmids		
pCPF-1	pCYB1 *tdcE*^+^ Amp^R^	Falke et al., 2016
pASK-IBA5	Amp^R^, expression vector	IBA Technologies
pfocA5	pASK-IBA5 *focA*^+^ with N-terminal Strep-II tag	Falke et al., 2010
pfocA5H284	Like pfocA5 but including UAA stop codon at codon 285 (lacking H285)	This study
pfocA5D283	Like pfocA5 but including UAG stop codon at codon 284 (lacking H284 and H285)	This study
pfocA5N282	Like pfocA5 but including UAA stop codon at codon 283 (lacking D283, H284, and H285)	This study
pfocA5E281	Like pfocA5 but including UAG stop codon at codon 282 (lacking N282, D283, H284, and H285)	This study
pfocA5R280	Like pfocA5 but including UAA stop codon at codon 281 (lacking E281, N282, D283, H284, and H285)	This study
pfocAL279	Like pfocA5 but including UGA stop codon at codon 280 (lacking R280, E281, N282, D283, H284, and H285)	This study
pfocAY278	Like pfocA5 but including UAA stop codon at codon 279 (lacking L279, R280 E281, N282, D283, H284, and H285)	This study


Growth of strains for analysis of β-galactosidase enzyme activity, for determination of formate levels excreted into the growth medium, or for hypophosphite-sensitivity analysis was generally performed in M9-minimal medium (Sambrook et al., 1989). The medium included 47.6 mM Na_2_HPO_4_ × 2 H_2_O, 22 mM KH_2_PO_4_, 8.4 mM NaCl, 20 mM NH_4_Cl, 2 mM MgSO_4_, 0.1 mM CaCl_2_, 0.1 mM thiamin dichloride, 1 mM trace element solution (Hormann and Andreesen, 1989) and 0.4% (w/v) glucose.

Growth of BL21(DE3) strains carrying plasmids encoding FocA variants for protein purification was done in TB medium, which included 1.2% (w/v) tryptone, 2.4% (w/v) yeast extract, 0.4% (w/v) glycerol, 0.4% (w/v) glucose, 100 mM potassium phosphate, pH 7, 2 mM MgSO_4_ and 0.37% (w/v) aspartic acid, pH 4, as described (Doberenz et al., 2014; Hunger et al., 2014). When required, antibiotics were used at a final concentration of 12.5 μg ml^-1^ for chloramphenicol, 50 μg ml^-1^ for kanamycin and 125 μg ml^-1^ for ampicillin.

### Hypophosphite-Sensitivity Test

The sensitivity of strains transformed with plasmids encoding various FocA variants toward the formate analog hypophosphite was tested essentially as described by Suppmann and Sawers (1994). Strains were grown anaerobically at 37°C in M9 minimal medium containing 0.4% (w/v) glucose and with 0.5 mM sodium hypophosphite. The growth rates of exponential-phase cells were compared after growth with and without added hypophosphite. Experiments were performed in triplicate.

### Construction of Plasmids

Expression plasmid pfocA5 (Falke et al., 2010) was used as the template for site-directed mutagenesis experiments using the oligonucleotides listed in Supplementary Table S1 and the Quik-Change II site-directed mutagenesis kit as described by the manufacturer (Agilent Technologies). Plasmid pfocA5Y278 was constructed by amplifying the *focA* gene using primers FocA5IBAfor and FocA5Y278rev (Supplementary Table S1) using pfocA5 plasmid DNA as template. Wild type FocA and all truncated variants carried a N-terminal Strep-tag and the plasmids were named according to the final C-terminal amino acid in the construct, e.g., pfocA5H284 had a stop codon introduced immediately after the codon encoding histidine residue at position 284. The plasmids encoding truncated variants of FocA were transformed into the indicated strain listed in **Table 1**, and which carried a chromosomal λ*fdhF*::*lacZ* transcriptional fusion (Falke et al., 2010).

### Preparation of Cell Extracts and Membrane Fractions

In order to determine the amount of the different FocA variants associated with the membrane fraction, cells were grown anaerobically in 0.5 or 1 l cultures of buffered TYEP (1% w/v tryptone, 0.5% w/v yeast extract, 100 mM sodium phosphate) rich medium including 0.4% (w/v) glucose, pH 7 (Begg et al., 1977). When the cultures reached an OD_600 nm_ of 0.8, cells were harvested by centrifugation at 2990 *g* for 30 min at 4°C, suspended in 4 ml of 50 mM Tris-HCl, pH 8 buffer including 170 mM NaCl per g wet cell mass. DNase I (10 mg ml^-1^) and 4 mM phenylmethylsulfonyl fluoride (PMSF) was then added. Cells were disrupted on ice by sonication (30 W power for 3 min × 3 min with 0.5 s pulses). Unbroken cells and cell debris were removed by centrifugation for 45 min at 43,000 *g* at 4°C, and the supernatant was used as the crude cell extract. Membrane fractions were prepared by ultracentrifugation at 100,000 *g* for 1 h at 4°C (Hunger et al., 2014). The membrane pellet was suspended directly in 50 mM Tris-HCl pH 8.0 buffer including 150 mM NaCl. The protein concentration of the subcellular fractions was determined (Lowry et al., 1951) using bovine serum albumin as the standard.

### Overproduction and Purification of Strep-FocA and Variants

For overproduction of Strep–N-FocA and its truncated variants, cultures of *E. coli* BL21(DE3) containing pASK-IBA5focA or the appropriate pfocA-variant were grown aerobically at 30°C in 1 l of TB medium supplemented with 125 mg l^-1^ ampicillin to an OD_600 nm_ of approximately 0.4. Gene expression was induced by addition of 0.2 mg l^-1^ anhydrotetracycline and cultures were incubated at 30°C with continuous shaking for a further 14 h. Cells were harvested by centrifugation at 2990 *g* for 30 min and 4°C, suspended in 10 ml of 50 mM Tris-HCl, pH 8.0 and 170 mM NaCl containing DNase I (10 mg ml^-1^) and 4 mM PMSF. Cells were disrupted on ice by sonication and the crude cell extract and membrane fractions were prepared as described above.

To solubilize FocA from the membrane 4 mg of *n*-dodecyl-β-D-maltoside (DDM) (Glycon Biochemicals, Luckenwalde, Germany) were added per milligram of protein under gentle stirring and incubation was continued at 4°C overnight. The solution was subsequently centrifuged for 1 h at 100,000 *g* and at 4°C. The resulting supernatant containing solubilized Strep-tagged FocA or its variants was supplemented with 3 nM avidin and was then loaded onto a 1 ml column containing a Strep-Tactin-Sepharose matrix (IBA, Göttingen). Further purification steps were carried out exactly as described in the IBA standard protocol under aerobic conditions at 4°C. The yield of Strep-N-FocA and the variants was generally approximately 1.5 mg l^-1^ of culture. Strep-N-FocA was then buffer-exchanged into 50 mM Tris-HCl, pH 8, including 150 mM NaCl, 2 mM DDM.

### Interaction Studies between FocA Variants and TdcE

To identify possible interaction between N-terminally Strep-tagged FocA variants and TdcE, we used a pull-down assay based on the IMPACT system (New England Biolabs). For overproduction of the (chitin-binding domain) CBD-intein-TdcE fusion protein (Falke et al., 2016), cultures of *E. coli* BL21 (Δ*act*) containing the expression plasmid pCPF-1 were grown aerobically in 1 l LB medium supplemented with 25 μg/ml chloramphenicol at 30°C with continuous shaking to an OD_600_ of approximately 0.5. Gene expression was induced by adding 0.4 mM IPTG to the culture and incubation was continued at 30°C for a further 3 h. After harvesting the cells by centrifugation, the wet cell paste was suspended at a ratio of 1 g per 3 ml in 50 mM Tris-HCl (pH 8,0), 170 mM NaCl, 5 μg DNase ml^-1^ and 0.2 mM PMSF. Cells were disrupted aerobically by sonication (Sonopuls Bandelin, Berlin) using a KE76 tip, 30 W power and 3 cycles of 4 min treatment with 0.5 s intervals. Unbroken cells and cell debris were removed by centrifugation for 30 min at 15,000 *g* at 4°C. Aliquots (2 mg) of this crude extract containing the overproduced CBD-intein-TdcE fusion protein were incubated with 100 μg of the purified FocA variants for 2 h at 4°C in a final volume of 1 ml. Subsequently, 100 μl chitin sepharose matrix was added and the samples were centrifuged for 5 min at 10,000 *g* at 4°C. The supernatant was discarded and the matrix was suspended in 1 ml 50 mM Tris-HCl (pH 8,0), 170 mM NaCl. This procedure was repeated twice and bound TdcE was eluted by suspending the matrix in 100 μl of buffer containing 50 mM dithiothreitol (DTT) as described (Falke et al., 2016). Afterward 20 μl of the elution fraction were analyzed by western blotting and interacting proteins were detected using either anti-PflB antibodies, which recognize TdcE (Falke et al., 2016), or anti-FocA antibodies.

### Polyacrylamide Gel Electrophoresis (PAGE) and Immunoblotting

Aliquots of 4 μg of the various purified, truncated FocA variants were separated by 12.5% (w/v) sodium dodecyl sulfate (SDS)-PAGE (Laemmli, 1970) and transferred to nitrocellulose membranes as described (Towbin et al., 1979). Affinity-purified antibodies directed against full-length FocA were used at a dilution of 1:3000 (Falke et al., 2010). Anti-Strep-tag II antibodies (IBA Biotechnology, Germany) were used at a dilution of 1:100,000. The secondary antibody conjugated to horse-radish peroxidase (Bio-Rad, Munich, Germany) was used according to the manufacturer’s instructions. Visualization of the antibody–antigen interaction was achieved using the enhanced chemiluminescent reaction (Agilent Technologies).

Blue-native (BN)-PAGE was performed with 5–13.5% (w/v polyacrylamide) gradient gels according to (Schägger and von Jagow, 1991) as described (Falke et al., 2010).

### Circular Dichroism (CD) Spectroscopy

Far-UV CD spectra were recorded on a Jasco J710 spectropolarimeter (Falke et al., 2010). Spectra of purified Strep-tagged FocA and Strep-tagged FocA variants (0.07 mg ml^-1^) were recorded in 20 mM Tris-HCl, pH 8.0, 150 mM NaCl, 0.2 mM EDTA, and 1 mM DDM at 20°C in a 0.1 cm cuvette. The α-helical content for FocA and its truncation variants based on the CD-spectral properties was determined using the program CDNN (Böhm et al., 1992).

### β-Galactosidase Activity Assays

The β-galactosidase enzyme activity was determined and calculated according to Miller (1972) as described (Falke et al., 2010). Each experiment was performed three times independently, and the activities for each sample were determined in triplicate.

## Results

### Membrane Integrity of C-terminally Truncated FocA Variants

**FIGURE 2:**
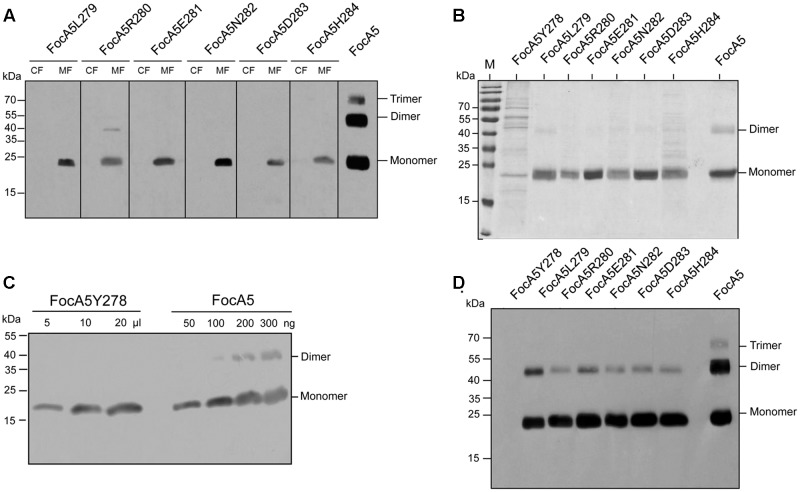
Denaturing SDS-PAGE analysis of FocA variants. **(A)** Western blot: Aliquots of cytoplasmic fractions (CF; 50 μg total protein) and membrane fractions (MF 50 μg total protein) were separated by SDS-PAGE (12.5% w/v polyacrylamide) and, after transfer to nitrocellulose, the blot was challenged with anti-FocA antiserum. **(B)** Coomassie-stained gel: Purified FocA variants (8 μg of protein, except FocA5Y278 where only 0.2 μg of protein could be obtained) were separated in a 12.5% (w/v) polyacrylamide SDS gel and stained with Coomassie Brilliant Blue. **(C)** Western blot: Estimate of the amount of the purified FocA5Y278 variant directly after elution from the Strep-Tactin affinity chromatography column. **(D)** Western blot: The same samples (1 μg of protein, except FocA5Y278 had only 0.2 μg of protein) applied to the gel shown in **(B)** were challenged with anti-FocA antiserum. The migration positions of molecular mass markers are shown on the left of each panel and the monomeric, dimeric, and trimeric forms of the FocA variants are shown on the right of the panels.

Based on the structural analysis of FocA proteins (Wang et al., 2009; Waight et al., 2010; Lü et al., 2011, 2012b; Czyzewski and Wang, 2012), the cytoplasmically oriented C-terminus forms part of a short α-helix that caps, but is not part of, the sixth transmembrane helix, probably stabilizing the protomer in the membrane (**Figure 1A**). For FocA-like FNTs the sixth TM helix is terminated by the conserved amino acid residues I/L-Y-L-R/K (amino acids 277–280 based on *E. coli* FocA numbering) followed by a variable number of amino acids (see **Figure 1B**). In FocA from *E. coli* five amino acids, with the sequence ENDHH, follow R280. To determine whether this C-terminal pentapeptide has a role in formate translocation or integrity of the FocA pentamer within the membrane, we generated variants with successive single amino acid truncations at the C-terminus (**Figure 1C**). All seven variants were overproduced in *E. coli* strain BL21 (DE3) and their biophysical properties were first compared with those of full-length, N-terminally Strep-tagged FocA (**Figure 2**). After sub-cellular fractionation, six of the seven truncation variants were shown to be membrane-associated (**Figure 2A**) and based on western blot analysis with antibodies against full-length FocA they migrated in SDS-PAGE with a molecular mass of approximately 22 kDa, which is characteristic for the aberrant migration of native and highly hydrophobic FocA (Suppmann and Sawers, 1994; Falke et al., 2010). The FocA truncation variant that could not be detected in the membrane fraction by western blotting was FocA5Y278, which lacked seven C-terminal amino acids, including the Y-L-R motif (**Figures 1A,C**).

The membrane fractions containing the different over-produced FocA variants were solubilized and each variant was affinity-purified using a Strep-Tactin matrix. Aliquots of each isolated protein were separated by SDS-PAGE and stained with Coomassie Brilliant Blue (**Figure 2B**). Only low amounts of FocA5Y278 could be isolated and the sample contained many contaminating polypeptides. While for all other FocA variants, including the native protein, approximately 1.5 mg of protein per l of culture could be isolated, only approximately 0.5–1% of this amount was obtained for the FocA5Y278 variant (**Figure 2C**); no evidence for the dimeric species could be observed for FocA5Y278. The dimeric and occasionally trimeric species are observed during SDS-PAGE due to incomplete denaturation because of the necessity of use a low denaturation temperature to prevent FocA self-aggregation during electrophoresis (Suppmann and Sawers, 1994; Hunger et al., 2014; see Materials and Methods). In contrast to FocA5Y278, all of the other six FocA variants could be isolated, with minor exceptions, in relatively pure form. In all cases traces of the dimeric species of each protein could be discerned, which was confirmed by western blot analysis (**Figure 2D**). The amount of the dimeric species for the truncated variants was significantly lower than was observed for the native protein (Falke et al., 2010; Hunger et al., 2014).

**FIGURE 3:**
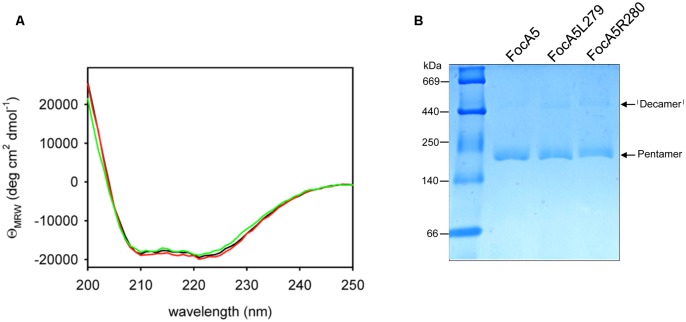
The secondary and quaternary structural features of native FocA5 are retained in the truncation variants FocA5L279 and FocA5R280. **(A)** Far-UV CD spectra were recorded at 20°C and at a protein concentration of 0.07 mg ml^-1^. The spectra are of FocA5 (black line), FocA5L279 (green line) and FocA5R280 (red line). The data are presented as molar ellipticity values per amino acid residue (ΘMRW). **(B)** Blue-native (BN) polyacrylamide gel electrophoretic analysis of FocA variants. Aliquots (5 μg of protein) of the indicated FocA variants were separated in a 5–13.5% gradient gel. The migration positions of molecular mass markers are shown on the left of the panel and the migration positions of pentameric and decameric forms of FocA are indicated on the right. The markers (Amersham) were thyroglobulin 669 kDa, ferritin 440 kDa and catalase 250 kDa, lactate dehydrogenase 140 kDa, and albumin 66 kDa.

To determine whether the truncated variants retained their α-helical characteristics and their pentameric quaternary structure, aliquots of each were analyzed by CD-spectroscopy (**Figure 3A**) and separated in blue-native (BN)-PAGE (**Figure 3B**). The spectra of FocA variants FocA5L279 and R280 were indistinguishable from native FocA5, and showed the twin troughs at 208 and 220 nm (**Figure 3A**), which are characteristic for the high α-helical content of FocA (Falke et al., 2010). The calculated α-helical content of the native protein was 66.4%, while that of FocA5L279 was 63.3% and of FocA5R280 67.8%. This indicates that the amino acid truncations resulting in FocA terminating with amino acid residue L279 had no significant impact on the secondary structure of the protein. Native FocA5, and the variant FocA5L279 and FocAR280 also migrated as pentameric species in BN-PAGE (**Figure 3B**), demonstrating that trunctions up to amino acid position 279 did not affect the quaternary structure of FocA in the membrane. Occasionally, what we interpret to be a ‘decameric’ species is observed for all three proteins shown in **Figure 3B**. This species has been observed previously (Hunger et al., 2014), and is presumed to be a dimer of pentamers; it was also observed for FocA5N282 and FocA5H284 (Supplementary Figure S1). No pentameric species for variant FocA5Y278 could be observed, suggesting that removal of L279 destabilized helix 6 in the membrane, possibly impeding oligomerization of the protein. Together, these data indicate that all seven C-terminally truncated FocA variants were membrane-associated and all but the shortest variant (FocA5Y278) formed pentamers like the native, full-length protein.

### Formate Export Was Only Negatively Impacted by Removal of a C-terminal Hexapeptide Including R280

**FIGURE 4:**
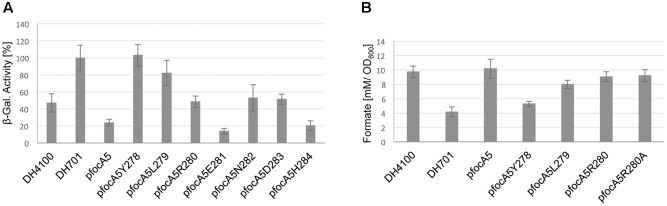
Analysis of formate export by strain DH701 (Δ*focA*) carrying different FocA C-terminal truncation variants. **(A)** The relative β-galactosidase activity (%) from a single-copy, formate-responsive *fdhF’-’lacZ* transcriptional fusion in *E. coli* strain DH701 (Δ*focA*) carrying the indicated plasmid derivatives after anaerobic growth in M9-glucose minimal medium is shown (see also Materials and Methods). 100% enzyme activity corresponded to 540 ± 140 units. DH4100 (wild type) acted as the positive control and DH701 (Δ*focA*) as negative control. **(B)** Formate levels in the culture medium of strains grown in M9-glucose minimal medium to an OD_600 nm_ of 0.4 were determined and are presented as mM formate per OD unit.

Strain DH701 carries a chromosomal mutation that prevents synthesis of FocA but the mutant is unaffected in PflB synthesis so that intracellular formate generation by the strain is unimpaired (Suppmann and Sawers, 1994; Hunger et al., 2014). The strain also has a single copy of a formate-responsive *fdhF-lacZ* reporter fusion allowing assessment of the impact of plasmid-encoded FocA variants on intracellular formate levels (Hunger et al., 2014). After anaerobic growth in M9 minimal medium with glucose as a carbon source, strain DH701 had a β-galactosidase enzyme activity of 540 ± 140 units, which was set as 100% (**Figure 4A**). In comparison, strain DH4100, which is an isogenic wild-type strain (Hunger et al., 2014), had a β-galactosidase enzyme activity that was approximately 45% of this value (**Figure 4A**). This result is in accord with FocA-dependent export of formate by strain DH4100, while the *focA* mutant DH701 accumulated formate intracellularly (Suppmann and Sawers, 1994; Doberenz et al., 2014; Hunger et al., 2014). Introduction of N-terminally Strep-tagged FocA encoded on plasmid pfocA5 into strain DH701 resulted in a β-galactosidase enzyme activity that was almost 80% reduced compared to the level in the original *focA* mutant (**Figure 4A**). The FocA variant truncated up to, and retaining, amino acid residue R280 exhibited β-galactosidase enzyme activity similar to that of DH4100 or to DH701 complemented with pfocA5 (**Figure 4A**). Variants with either one (FocA5H284) or four (FocA5E281) amino acid residues removed from the C-terminus exhibited very low expression levels, indicating that these strains have very low intracellular formate levels (**Figure 4A**). Why these variants exported more formate than the other three (FocA5D283, N282 and R280), which had β-galactosidase enzyme activities similar to that of DH4100, is currently unclear. Nevertheless, these results indicate that none of these five truncated variants was impaired in its ability to export formate.

Additional removal of residue R280 (variant FocAL279), however, reduced the β-galactosidase enzyme activity to a level intermediate between that of the wild type and the *focA* mutant (**Figure 4A**). Finally, removal of amino acid residue L279 yielded a variant (FocAY278) that had a β-galactosidase enzyme activity similar to that of the *focA* mutant DH701. The combined results shown in **Figures 2–4** indicate that the amino acid residue leucine 279 is essential for both pentamer stability and for formate translocation activity, while arginine 280 is necessary only to confer optimal formate export activity upon FocA.

**FIGURE 5:**
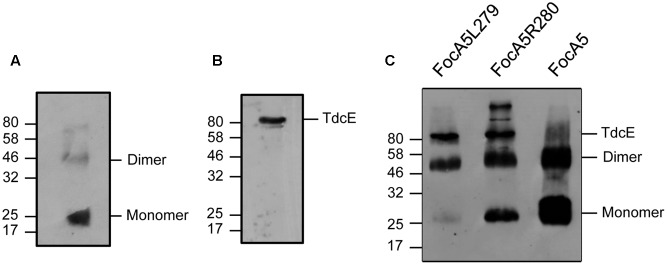
Pull-down assay showing the interaction between selected truncated FocA variants and the PflB homolog TdcE. The pull-down assay was performed as described in the section “Materials and Methods.” An aliquot (2 mg) of soluble fraction derived from BL21(DE3)/ pCPF-1 cells containing an over-produced chitin-binding protein-TdcE fusion was mixed with 100 μg of purified native FocA5 **(A)** or the truncation variants FocA5L279 or FocA5R280 **(C)**. Western blots using anti-FocA antibodies **(A)**, anti-PflB antibodies **(B)**, which interact with TdcE (Falke et al., 2016), and a mixture of anti-FocA + anti-PflB antibodies **(C)** are shown. In the blot shown in **(C)**, 3 μg of purified native FocA5 was applied to the gel as a control. The migration positions of TdcE and of the monomeric and dimeric forms of FocA are indicated on the right of the respective panels. The molecular mass standards (New England Biolabs) are indicated in kDa.

Recent studies have shown that an interaction between FocA and PflB is necessary to allow the channel to export formate efficiently (Doberenz et al., 2014). FocA also interacts with TdcE, which is a glycyl radical enzyme that is functionally and structurally homologous to PflB (Falke et al., 2016). To determine whether an impaired interaction with PflB/TdcE was the reason why the FocA5L279 variant, which includes L279 as the C-terminal residue, had reduced formate-translocating activity, we examined the ability of this derivative to interact specifically with TdcE fused to the chitin-binding domain in a pull-down assay using chitin beads (**Figure 5**; see Materials and Methods). The interaction between TdcE and the FocA5L279 and the FocA5R280 variants was not impaired by the truncations, indicating that removal of the C-terminal amino acids of FocA did not affect the ability of the protein to interact with the TdcE/PflB family of proteins.

### Formate Levels in the Culture Medium

As an independent assessment of formate translocation by the truncated FocA variants, formate levels in the growth medium were determined after fermentative growth with glucose. Nearly 10 mM formate per unit of OD_600 nm_ accumulated in the culture medium after 2.5 h growth of the wild-type strain DH4100 (**Figure 4B**). In contrast, the *focA* mutant DH701 exported 60% less formate into the culture medium over the same time period. This finding correlates with the increase in the relative concentration of formate inside the cells of strain DH701 as determined by the measurement of the activity of the formate-responsive *fdhF-lacZ* reporter fusion (**Figure 4A**). Introduction of plasmid-encoded, N-terminally Strep-tagged FocA into the *focA* mutant restored the level of formate accumulation in the culture medium to wild-type levels (**Figure 4B**). The truncation derivative FocA5R280, lacking five C-terminal amino acid residues, also exhibited near-wild-type levels of activity, as did a variant of this construct in which residue arginine 280 was converted to an alanine (**Figure 4B**). This suggests that the positive charge conferred by the arginine was not important for retention of formate export function of this truncated FocA variant. Truncation variants in which fewer than six amino acid residues were removed from the C-terminus showed wild-type levels of formate in the culture medium (data not shown).

Removal of R280 (FocA5L279 variant) resulted in a 20% reduction in formate levels in the culture medium relative to the wild type, while removal of L279 (FocA5Y278 variant) reduced the formate level by half compared with the wild type (**Figure 4B**). These results are broadly in agreement with the parallel increase in intracellular formate levels determined using the formate reporter (**Figure 4A**).

### Minimally Six C-terminal Amino Acid Residues Are Necessary for Formate Import through FocA

**FIGURE 6:**
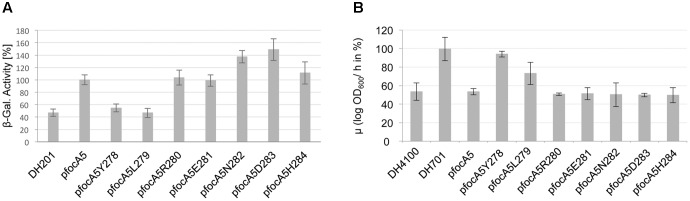
Analysis of formate import by strain DH201 (Δ*focA-pflB*) carrying different FocA C-terminal truncation variants. **(A)** The relative β-galactosidase activity (%) from a single-copy, formate-responsive *fdhF’-’lacZ* transcriptional fusion in *E. coli* strain DH201 (Δ*focA-pflB*) carrying the indicated plasmid derivatives after anaerobic growth in M9-glucose minimal medium is shown (see also Materials and Methods). 100% enzyme activity corresponded to 1150 ± 140 units. DH201 (Δ*focA-pflB*) acted as negative control. **(B)** The relative growth rates (μ) of the indicated strains were determined after anaerobic growth in M9-glucose minimal medium with or without 0.5 mM sodium hypophosphite. The 100% value corresponds to the growth rate of DH701 (Δ*focA*) in the absence of hypophosphite divided by the growth rate in the presence of hypophosphite.

FocA-dependent formate uptake can also be assessed using the *focA-lacZ* reporter fusion by measuring β-galactosidase enzyme activity after growth of strain DH201, which carries a deletion in the *focA-pflB* operon (Hunger et al., 2014). This strain is a derivative of RM201 (Δ*focApflB*) and is incapable of synthesizing formate via pyruvate breakdown (Sawers and Böck, 1988). Consequently, when DH201 is grown anaerobically with glucose as carbon source no β-galactosidase enzyme activity can be measured (Hunger et al., 2014). However, addition of formate exogenously to the growth medium results in inefficient formate uptake by DH201 through an as yet uncharacterized second uptake system (**Figure 6A**; Suppmann and Sawers, 1994; Hunger et al., 2014). Introduction of the native *focA* gene on plasmid pfocA5 into DH201 resulted in a 2.5-fold increase in β-galactosidase enzyme activity (**Figure 5A**). While both truncated FocA variants with deletions of seven (pfocA5Y278) and six (pfocA5L279) C-terminal amino acid residues had β-galactosidase enzyme activities similar to DH201, all other truncations in which fewer amino acid residues were removed had enzyme activities like the native, full-length channel (**Figure 6A**).

The formate analog hypophosphite is an inhibitor of the PflB reaction and is translocated into anaerobically growing *E. coli* cells by FocA (Suppmann and Sawers, 1994). Mutants lacking PflB show a growth restriction compared with the wild type when growing fermentatively with glucose (Suppmann and Sawers, 1994) and a similar growth restriction is observed for the wild type during glucose fermentation when high concentrations of hypophosphite are added to the medium; *focA* mutants thus have an anaerobic growth phenotype like a *pflB* mutant (Suppmann and Sawers, 1994; Beyer et al., 2013). Here, we modified the hypophosphite assay to measure changes in anaerobic growth rate (μ) when the culture was supplemented with 0.5 mM hypophosphite compared with when no hypophosphite was added (**Figure 6B**). The μ of the *focA* mutant DH701 was double that of the wild type DH4100, regardless of whether hypophosphite was added or not, but when pfocA5 encoding Strep-tagged FocA was introduced into DH701, the growth rate of the strain was reduced to a level similar to that of DH4100 in the presence of hypophosphite (**Figure 6B**). Progressive single amino acid residue shortening of the FocA C-terminus revealed that removal of the pentapeptide E281-N282-D283-H284-H285 had no impact on the hypophosphite sensitivity of the variants (**Figure 6B**), whereby they all had the same slow-growth phenotype of DH4100 or DH701/pfocA5, which both synthesize full-length FocA. However, removal of the C-terminal hexapeptide including R280 improved the growth rate significantly, indicating reduced uptake of hypophosphite. Removal of the next amino acid L279 resulted in similar rapid growth compared to the *focA* mutant DH701 (**Figure 6B**). These findings paralleled those observed for uptake of formate by these truncated FocA variants and indicated that the 5 C-terminal amino acid residues are non-essential for a fully functional FocA.

## Discussion

Like the N-terminus, the C-terminus of FNT channel proteins is localized in the cytoplasm. The number of amino acid residues attached to the final transmembrane helix as it emerges from the membrane varies in length depending on the member of the FNT superfamily. For FocA-like proteins, including archaeal FdhC, FocB from *E. coli* and FocA from *Vibrio cholerae*, there is a reasonably well-conserved tripeptide with the sequence Y-L-R/K directly at the C-terminus, after it emerges from the cytoplasmic membrane (**Figures 1A,B**). This amino acid sequence at the end of transmembrane helix 6 has the characteristics of a membrane-aqueous phase interfacial anchoring peptide (Killian and von Heijne, 2000). The basic residue at position 280 (*E. coli* numbering) is frequently followed by an acidic residue. The C-termini of the HSC and NirC channels are somewhat longer and appear to have different sequence requirements. In particular, a conserved and highly charged C-terminal region (approximately 20 amino acids in length) in the nitrite transporter NitM from marine α-cyanobacteria negatively regulates nitrite transport by the bacteria (Maeda et al., 2015). This finding suggests that the C-terminus of NitM binds a regulator protein or the C-terminus itself has a structural function in controlling nitrite transport.

We show in this study that for FocA from *E. coli* all C-terminal amino acids can be removed up to, but not including, the basic residue at position 280 without impinging on either the oligomeric integrity or the functionality of FocA in import or uptake of formate. This is in accord with the cytoplasmic C-terminus of FocA from *V. cholerae* being very short, terminating with a glutamate after the lysine, which is a conservative substitution for R280 of *E. coli* FocA in the Y-L-R/K motif (Waight et al., 2010). Removal of the hexapeptide including R280 did not affect either the α-helical content of FocA, as judged by CD-spectroscopy, or the ability to form a pentamer in the membrane; however, import of formate or the formate analog hypophosphite was abolished in the variant (see **Figure 6**). Formate export was still partially functional in the FocA5L279 variant. These results substantiate earlier findings with other FocA amino acid variants (Hunger et al., 2014) that clearly indicated formate export through the channel had different requirements compared to formate import.

A key difference between the two mutant strains of *E. coli* used in this study was that, in contrast to DH701, strain DH201 lacked PflB, the enzyme that is responsible for intracellular formate generation. Both PflB and its functional homolog TdcE, have been shown to interact with FocA and promote formate uptake through the channel (Doberenz et al., 2014; Falke et al., 2016). Both PflB and TdcE are present in either an enzymatically active or inactive form, depending on the metabolic status of the anaerobic cell (Wagner et al., 1992; Sawers and Clark, 2004). We have shown previously that when PflB is present in its inactive species it impedes formate uptake by FocA (Doberenz et al., 2014). Similarly, when the enzyme is absent formate uptake via FocA is significantly less efficient than when PflB is present and active. Active PflB is present in strain DH701 and thus likely promotes formate export by FocA, even if the C-terminal peptide including R280 is removed. Notably, we could demonstrate clearly by *in vitro* pull-down studies that TdcE still interacts efficiently with FocA5L279. Because TdcE is synthesized only at very low levels when *E. coli* is grown with glucose (Sawers et al., 1998), the above finding supports the notion that the absence of PflB is likely to be the reason why no formate uptake in strain DH201 encoding FocA5L279 occurs but when it is present in strain DH701 formate can still be exported but at reduced levels compared with native FocA. While the mechanism underlying this difference in activity remains to be established, these findings might have relevance to the previously observed structural interaction that can occur between the C- and N-termini of FocA from *Salmonella typhimurium* (Lü et al., 2011). The interaction leads to stabilization and ordering of the Ω-loop (Lü et al., 2011), which in turn is important in directing formate passage through the channel (Waight et al., 2013; Hunger et al., 2014). Disruption of the interaction between the C- und N-termini in the FocA5L279 truncation variant might explain the loss of formate translocating activity.

Removal of L279 strongly affected the stability of FocA in the membrane and it was not possible to demonstrate a pentameric form of the protein. This result is in accord with the transmembrane helix-stabilizing function of leucine followed by a positively charged residue at the membrane-cytoplasm interface (Killian and von Heijne, 2000). The C-terminal tyrosine in the FocA5Y278 variant is clearly insufficient on its own to anchor the transmembrane helix to deliver a functional FocA protein.

While the cytoplasmic C-terminal peptides after the Y-L-R/K residues of FocA-like channels belonging to the FNT family are comparatively short and appear not to have an essential role in FocA function, those of the nitrite channel NirC and the lactate transporter PfFNT from *P. falciparum* are significantly longer by between 18 and 25 residues, respectively, than that of *E. coli* FocA. The C-terminus of the NitM nitrite transporter in some cyanobacteria clearly regulates substrate translocation (Maeda et al., 2015). Therefore, it will be important to determine the apparently distinct mechanisms underlying these regulatory processes within the FNT protein family.

## Author Contributions

DH, MR, DF, and HL: Design, analysis and execution of the experiments, and drafting of the manuscript. RGS: Data analysis and interpretation and drafted the manuscript.

## Conflict of Interest Statement

The authors declare that the research was conducted in the absence of any commercial or financial relationships that could be construed as a potential conflict of interest.
