# Supplementary material for: The C-terminal Six Amino Acids of the FNT Channel FocA Are Required for Formate Translocation But Not Homopentamer Integrity
Source: Front Microbiol. 2017 Aug 22;8:1616. doi: 10.3389/fmicb.2017.01616 (PMC5572259; doi:10.3389/fmicb.2017.01616)
Supplement: Supplementary file 1 [file Image_1.pdf]

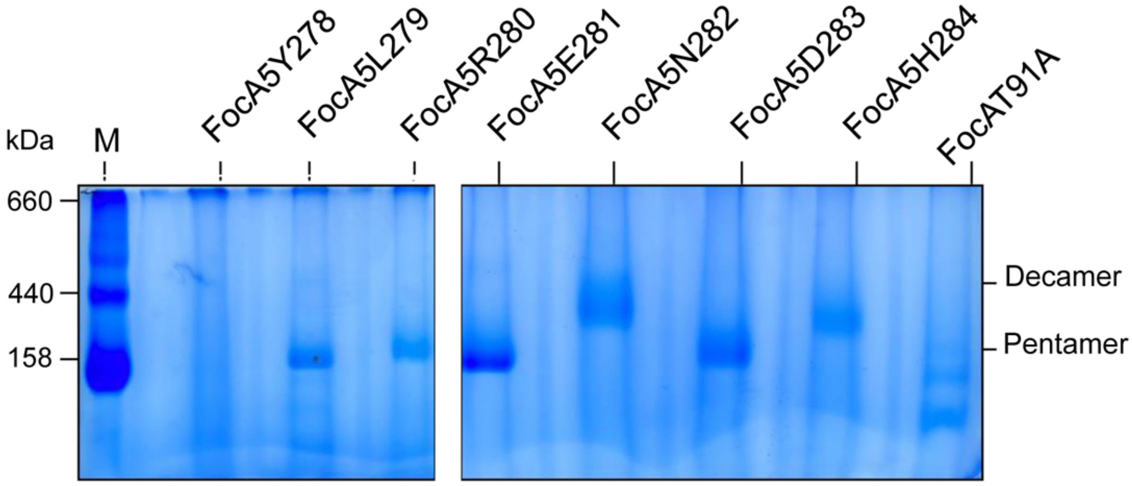

Figure S1. Blue-native (BN) polyacrylamide gel electrophoretic analysis of FocA variants. Aliquots (5 μg of protein) of the indicated FocA variants were separated in a 5–13.5 % gradient gel. The control was the variant FocAT91A, which upon migration disassembles into trimeric and dimeric species (Hunger et al., 2014). The migration positions of molecular mass markers are shown on the left of the Figure and the migration positions of pentameric and decameric forms of FocA are indicated on the right. The markers were thyroglobulin 660 kDa, ferritin 440 kDa and aldolase 160 kDa.

Reference

Hunger, D., Doberenz, C., and Sawers, R. G. (2014). Identification of key residues in the formate channel FocA that control import and export of formate. *Biol. Chem*. 395, 813-825.
