# Supplementary material for: The C-terminal Six Amino Acids of the FNT Channel FocA Are Required for Formate Translocation But Not Homopentamer Integrity
Source: Front Microbiol. 2017 Aug 22;8:1616. doi: 10.3389/fmicb.2017.01616 (PMC5572259; doi:10.3389/fmicb.2017.01616)
Supplement: Supplementary file 2 [file Table_1.pdf]

Table S1. Oligonucleotides used in this study.

Primer	Sequence 5' → 3'<br>Underlined codon indicates the substitution introduced	Encodes up to amino acid
FocA5IBAfor	ATG GTA GGT CTC AGC GCC AAA GCT GAC AAC<br>CCT TTT GAT CTT T	Y278
FocA5Y278 rev	ATGGTAGGTCTCATATCATTAGTAAATGACCCAGTATGTCAACCC	
FocA5L279 for	GTCATTTACCTGTGAGAAAACGACCAC	L279
FocA5L279 rev	GTGGTCGTTTTCTCACAGGTAAATGAC	
FocA5R280 for	ATTTACCTGCGTTAAAACGACCACCAT	R280
FocA5R280 rev	ATGGTGGTCGTTTTAACGCAGGTAAAT	
FocA5E281 for	TACCTGCGTGAATAGGACCACCATTGA	E281
FocA5E281 rev	TCAATGGAGGTCCTATTCACGCAGGTA	
FocA5N282 for	CTGCGTGAAAACTAACACCATTGATAT	N282
FocA5N282 rev	ATATCAATGGTGTTAGTTTTCCAGCAG	
FocA5D283 for	CGTGAAAACGACTAGCATTGATATCT	D283
FocA5D283 rev	AGATATCAATGCTAGTCGTTTTCACG	
FocA5H284 for	GAAAACGACCACTAATGATATCTAACT	H284
FocA5H284 rev	AGTTAGATATCATTAGTGGTCGTTTTC	
FocAR280Afor	GGTCATTTACCTGGCTGAAAACGACCAC	R280A
FocAR280Arev	GTGGTCGTTTTCAGCCAGGTAAATGACC	
